# Measuring proactive-instrumental aggression in adolescents and young adults—development and preliminary validity evidence for the Degree-of-Quiz-Difficulty Paradigm

**DOI:** 10.3389/fpsyg.2026.1876347

**Published:** 2026-09-03

**Authors:** Daniel Schleicher, Constanze Käss, Irina Jarvers, Angelika Ecker, Ricarda Jacob, Stephanie Kandsperger, Romuald Brunner

**Affiliations:** Department of Child and Adolescent Psychiatry and Psychotherapy, University of Regensburg, Regensburg, Germany

**Keywords:** adolescents, Degree-of-Quiz-Difficulty Paradigm, empathy, instrumental aggression, proactive aggression, prosocial behavior, reactive aggression, young adults

## Abstract

**Introduction:**

Aggression can be distinguished into reactive and proactive forms, which differ in their motivational and emotional characteristics. While reactive aggression is typically triggered by provocation and accompanied by heightened emotional arousal, proactive aggression is goal-directed, instrumentally motivated, and characterized by greater emotional control. Despite its relevance for antisocial behavior, proactive aggression is still assessed predominantly through self-report measures, and experimental paradigms specifically designed to capture it remain scarce, particularly for adolescents.

**Methods:**

The present study aimed to develop and provide preliminary validity evidence for a novel experimental paradigm, the Degree-of-Quiz-Difficulty Paradigm (DoQD), to objectively assess proactive aggression in adolescents and young adults. In this competitive task, participants (*N* = 54; *M*_age_ = 14.70, *SD*_age_ = 1.70; 12–19 years) assigned quiz difficulty levels to themselves and an alleged opponent, with the opponent’s assigned difficulty serving as an index of proactive aggression.

**Results:**

Although the difficulty index was not associated with self-reported proactive aggression, it showed the expected significant associations with caregiver-reported externalizing behaviors, particularly rule-breaking behavior, suggesting links to instrumental aggression. In contrast, no associations emerged with psychophysiological stress indicators, supporting the notion that proactive aggression occurs without heightened emotional arousal. Higher difficulty indices were also associated with increased feelings of guilt during and after the task, indicating that guilt may accompany but not necessarily inhibit proactive aggressive behavior. Regarding personality traits, the difficulty index showed a significant negative association with cooperativeness, whereas associations with empathy were not significant after correction for multiple testing.

**Discussion:**

Overall, the findings provide preliminary validity support for the DoQD as an objective, time-efficient behavioral paradigm relevant to the assessment of proactive aggression, with evidence limited to selected validity indicators.

## 1 Introduction

Aggression can be described as “attacking behavior” and can be functionally differentiated into a reactive and proactive component (Dodge and Coie, 1987). Reactive aggression is typically understood as an impulsive, anger- or fear-related response to perceived threat or frustration, serving to reduce internal tension and often reflecting deficits in emotion regulation (Berkowitz, 1969; Marsee and Frick, 2007; Vitaro and Brendgen, 2012). In contrast, proactive aggression is primarily goal-directed and driven by instrumental motivation, aiming at the attainment of desired outcomes rather than a response to provocation (Vitaro and Brendgen, 2005). Provocation therefore does not constitute the central trigger of proactive aggression. Instead, it is associated with resource acquisition, dominance, and the fulfillment of personal needs (Vitaro and Brendgen, 2012). Consequently, proactive aggression is typically characterized by a higher degree of emotional control, empathy dysfunction and deliberate planning (Blair, 2018). In general, both reactive and proactive aggressive behaviors tend to be more pronounced in males than in females (Mayberry and Espelage, 2007), and the two forms of aggression rarely occur independently of one another (Kempes et al., 2005). With regard to associations with personality and temperament traits, both aggression components are frequently found to be negatively correlated with facets such as agreeableness, conscientiousness, and indicators of character maturity, including self-directedness and cooperativeness (Fossati et al., 2009; Kerekes et al., 2017). Furthermore, the literature suggests that reduced cognitive and affective empathy is observed in adolescents exhibiting high levels of both reactive and proactive aggression (Euler et al., 2017).

The assessment of aggression differs substantially between reactive and proactive aggression. Reactive aggression can be assessed on multiple levels, including well-established self- and other-report questionnaires as well as a wide range of experimental paradigms that allow for a relatively objective assessment of aggressive behavior. Numerous experimental tasks are available for this purpose: Prominent examples include the Taylor Aggression Paradigm (TAP) (Taylor, 1967), the Point-Subtraction Aggression Paradigm (PSAP) (Cherek et al., 2003), and the Hot Sauce Paradigm (Lieberman et al., 1999), all of which rely on provocation and the measurement of aggressive responses to perceived threat or frustration. In contrast, proactive aggression is still assessed almost exclusively through self-report questionnaires. Experimental paradigms aiming to capture proactive aggression are rare. Only a limited number of studies have addressed its experimental assessment (Zhu et al., 2019), particularly in children and adolescents (e.g., Moore et al., 2018), despite evidence that externalizing behaviors peak during adolescence (Chi and Cui, 2020). Consequently, many studies rely on paradigms originally designed to measure reactive aggression and/or classify aggressive behavior in non-provoked trials as proactive (e.g., Dambacher et al., 2015; Moore et al., 2018). However, this approach has conceptual limitations, as core characteristics of proactive aggression (such as instrumental, goal-directed, and planned harmful behavior) may not be sufficiently represented.

More recent approaches have attempted to address these limitations by developing experimental paradigms specifically targeting proactive aggression. One of the few experimental tasks proposed for this purpose is the Reward Interference Task (RIT) (Zhu et al., 2019), which aims to minimize provocation by allowing participants to engage in instrumental, goal-directed interference in order to increase their own monetary reward. Although this paradigm represents an important step toward the experimental assessment of proactive aggression, feedback-related frustration, repeated loss experiences, and competitive elements resulting from the prior encounter between opponents may nevertheless elicit reactive aggressive processes, thereby limiting a clear attribution of observed behavior to proactive aggression. In addition, virtual reality–based paradigms have gained increasing attention, as they allow the simulation of complex and ecologically valid social scenarios in which a wide range of behaviors can be observed under controlled conditions. For example, participants may encounter situations in which their path is blocked, prompting aggressive behaviors (e.g., pushing a virtual character aside) in order to proceed (Lobbestael and Cima, 2021). However, such scenarios inherently involve elements of provocation or obstruction, which may trigger reactive rather than purely proactive aggression. Similar conceptual limitations apply to other paradigms frequently discussed in the context of proactive aggression. In the Tangram Help/Hurt Task (Saleem et al., 2015), aggressive behavior is operationalized via the assignment of difficult puzzles to another person. In this paradigm, tangram difficulty is limited to categorical distinctions (easy, medium, hard) rather than an objective, more fine-grained measure of task difficulty, and the harmful assignment is not directly tied to personal goal attainment. Paradigms originating from behavioral economics, such as the Lies in Disguise Paradigm (Fischbacher and Föllmi-Heusi, 2013), have also been considered in this context, but primarily capture strategic or immoral behavior (misreporting the outcome of a die roll) rather than aggression directed toward harming another individual.

In summary, despite considerable progress in the assessment of aggression in youth, important conceptual and methodological challenges remain regarding the assessment of specific forms of aggression (Evans et al., 2024). The assessment of proactive aggression has predominantly relied on subjective self-report measures. Experimental paradigms are comparatively scarce and are often adaptations of tasks originally developed to assess reactive aggression. As such, they frequently overlap with reactive components (e.g., responses to provocation or frustration), do not consistently incorporate the element of personal goal attainment, or fail to provide differentiated behavioral outcome measures. Moreover, many of these paradigms involve numerous experimental trials within highly artificial laboratory settings and validation in younger populations remains limited. Against this background, the aim of the present study was to develop and validate a paradigm for the objective assessment of proactive aggression in adolescents and young adults. The paradigm was designed to integrate established components of aggression paradigms (e.g., a competitive setting against an alleged opponent) while maintaining a time- and resource-efficient experimental design. Experimental aggression paradigms commonly rely on competitive performance situations in which participants can intentionally disadvantage an opponent to improve their own outcome, for example by administering aversive stimuli or impairing the opponent’s task performance (e.g., Taylor, 1967; Cherek et al., 2003; Boccadoro et al., 2021). Reviews have emphasized the value of behavioral laboratory paradigms for differentiating reactive and proactive aggression while highlighting the need for further development of such measures, particularly for proactive aggression (Polman et al., 2007; Hubbard et al., 2010).

Based on these considerations, we developed the Degree-of-Quiz-Difficulty Paradigm (DoQD), which builds on this behavioral principle by operationalizing the opportunity to disadvantage an opponent through the allocation of quiz difficulty and was intended to capture core features of proactive aggression. In the DoQD, participants are informed that they will compete in a quiz against another participant and that winning (i.e., answering more questions correctly) will result in an additional €10 voucher on top of the standard compensation, thereby operationalizing instrumental motivation and personal goal attainment. Participants are further told that they have been randomly selected to assign the quiz questions between themselves and the alleged opponent, a situation requiring deliberate and emotionally controlled decision-making. The sum of difficulty levels assigned to the other participant served as the behavioral index of competitive decisions relevant to proactive aggression. Using standardized quiz difficulty provides an objective behavioral measure of participants’ willingness to disadvantage an opponent for personal gain in a controlled laboratory setting. A higher difficulty index reflects a greater tendency to manipulate the study conditions for personal gain while accepting that the other person may face more challenging conditions—without prior provocation or frustration. A detailed description of the procedure is provided in Section 2.3.1.

To validate the paradigm, we hypothesized that the difficulty index would show positive associations with aggression scores in both self-reports provided by the participants and informant reports provided by their caregivers (i.e., parents or legal guardians), particularly with regard to proactive aggression components, as evidence of convergent validity. In contrast, we expected negative associations with personality and temperament traits related to empathy and cooperativeness, providing evidence of discriminant validity. In addition, changes in mood and heart rate were examined exploratorily to further characterize the behavioral responses during the task. Given the higher degree of emotional control typically associated with proactive aggression (compared to reactive aggression following provocation) we did not expect significant associations with psychophysiological stress responses.

## 2 Materials and methods

### 2.1 Study design

The study was a within-subject, cross-sectional experiment and was pre-registered in the German Register of Clinical Trials (DRKS00027335). It was conducted in accordance with the ethical principles outlined in the Declaration of Helsinki by the World Medical Association and received ethical approval from the Ethics Committee of the University of Regensburg on November 17, 2021 (reference: 21–2658-101). Written informed consent was obtained from all participants and, where applicable, their legal guardians. Participation was voluntary and could be withdrawn at any time.

### 2.2 Recruitment and participants

Based on previously reported correlations between empathy and proactive aggression, a medium effect size (*r* = 0.35) was assumed (e.g., Chen et al., 2021). An *a priori* power analysis with (1 − β) = 0.80 and α = 0.05 indicated a minimum required sample size of *N* = 46. The final sample comprised 54 adolescents and young adults (aged 12–19 years) from the general population without psychiatric disorders (*M*_age_ = 14.70, *SD*_age_ = 1.70; biological sex: 29 female, 25 male; gender identity: 28 female, 26 male). Participants were recruited via social media, email lists, and postings at schools and universities. A cover story was used during recruitment, describing the study as an investigation of gaming behavior in rating and quiz tasks and its associations with temperament and personality traits. Data were collected between February and June 2022 at the Department of Child and Adolescent Psychiatry and Psychotherapy, University of Regensburg, as well as at secondary schools representing different educational tracks. Inclusion and exclusion criteria were assessed during recruitment using a standardized checklist. Participants were required to have sufficient proficiency in German and to be between 12 and 19 years of age. Individuals with current or past psychiatric or psychotherapeutic treatment, or with any diagnosed psychiatric or neurological disorder, were excluded. Participants were informed that they would receive a €15 voucher for participation and could earn an additional €10 voucher if they won the quiz, as part of the experimental cover story. All participants ultimately received €25, regardless of performance.

### 2.3 Material

#### 2.3.1 Degree-of-Quiz-Difficulty Paradigm (DoQD)

The experimental paradigm was based on a cover story in which participants were told that they would take part in a quiz and compete against an unknown opponent. This opponent did not actually exist. No information was provided about the alleged opponent, and no interaction or introduction took place. This procedure was chosen to prevent the elicitation of reactive behaviors as well as (dis)liking or sympathy effects resulting from interpersonal confrontation.

**Figure 1:**
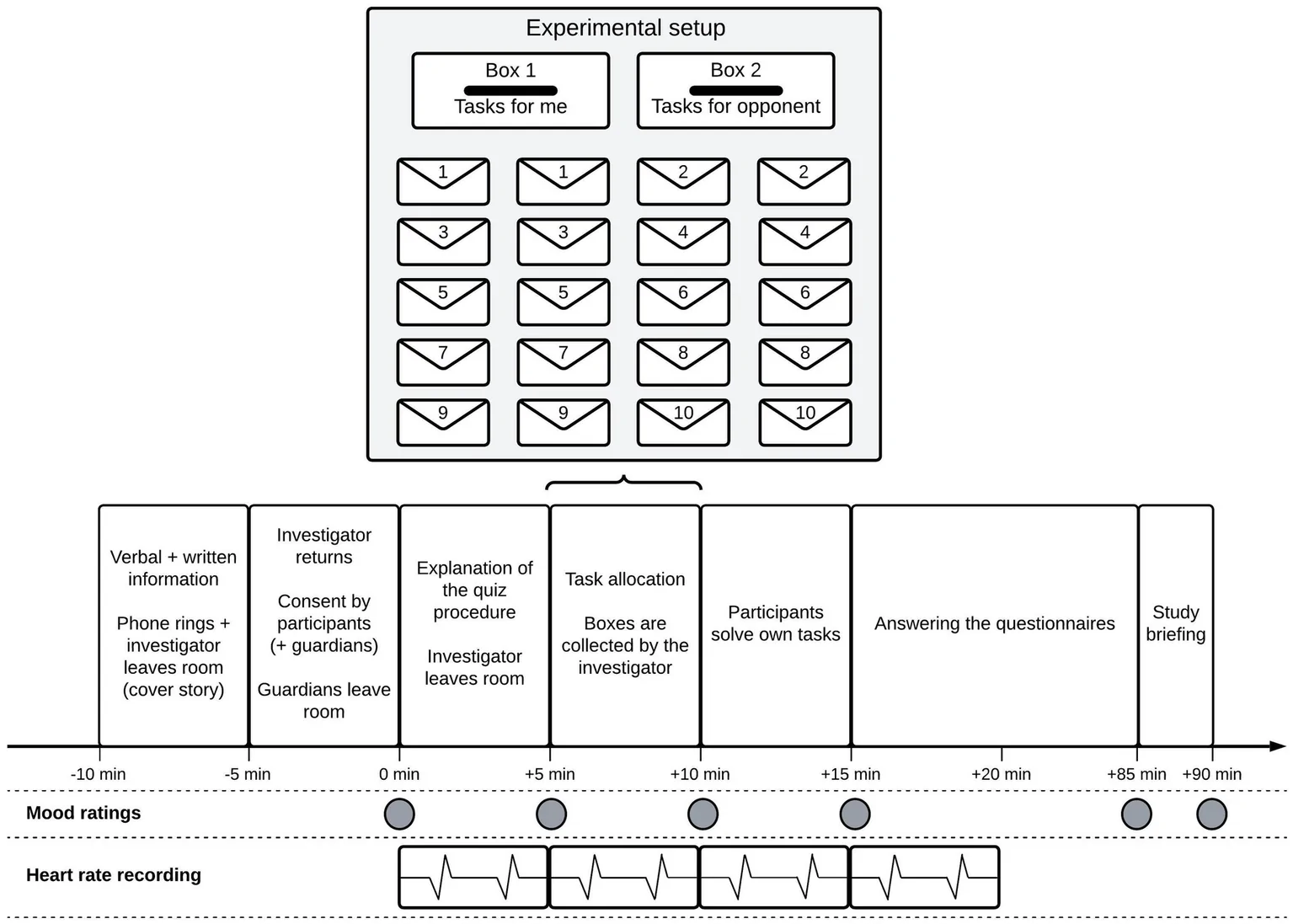
Overview of the procedure and experimental setup of the Degree-of-Quiz-Difficulty Paradigm.

At the beginning of the paradigm, participants were informed that they had been selected by random draw to assign the difficulty level of the quiz questions both for themselves and for the opponent. Each person was said to complete 10 quiz questions. In front of the participant were 20 sealed envelopes, each labeled with a number from 1 to 10, with each number appearing twice. In addition, two boxes labeled “Tasks for me” and “Tasks for the other person”, each with a slot for inserting envelopes, were placed in front of the participant (Figure 1). Participants were informed that the numbers on the envelopes represented the difficulty level of the tasks inside (1 = very easy, 10 = very difficult), although the quiz questions themselves could not be viewed in advance. Participants were further assured that the opponent would not be aware that the task assignment had been made by them and that the experimenter would not look into the boxes when transferring the envelopes to the opponent. It was additionally explained that the person who answered more questions correctly would receive an extra €10 voucher.

After the instruction, the experimenter left the room, and participants distributed the sealed envelopes between the two boxes. The experimenter then briefly collected the box designated for the alleged opponent. The difficulty index for the opposing participant ranged from 30 (minimum) to 80 (maximum), with a value of 55 reflecting an equal distribution of task difficulty. Participants subsequently opened the 10 envelopes they had assigned to themselves and completed the corresponding tasks. These tasks consisted of non-verbal matrix reasoning problems with increasing levels of difficulty, requiring participants to select the missing element to complete a visual matrix, thereby ensuring the credibility of the cover story. Finally, the completed tasks were collected by the experimenter for evaluation.

#### 2.3.2 Aggression

Aggression facets were assessed using the Differential Aggression Questionnaire (DAF) (Petermann and Beckers, 2014), which comprises 16 items on a 4-point scale (0 = never, 3 = often). It measures Anger-Aggression and Defensive Aggression Attribution (Reactive Aggression) as well as Resource Acquisition and Exercise of Power/Dominance (Proactive Aggression). The DAF shows satisfactory internal consistency, retest reliability, and validity, supported by coherent factor structures and meaningful correlations with related constructs (Petermann and Beckers, 2014).

The German Aggression Questionnaire (AQ) (Buss and Perry, 1992; Werner and von Collani, 2004) was used to assess Physical and Verbal Aggression (behavioral), Hostility/Distrust (cognitive), and Anger (affective). It consists of 29 items rated on a 4-point scale (1 = does not apply, 4 = strongly agree). Internal consistency, retest reliability, and differential validity are acceptable (Werner and von Collani, 2004).

Emotional and behavioral problems were measured with the Youth Self-Report (YSR/11–18R) and the corresponding caregiver-report form, the Child Behavior Checklist (CBCL/6–18R) (Döpfner et al., 2014). Both comprise 112 items rated on a 3-point scale (0 = not true, 2 = very true or often true). Both questionnaires show high reliability and their validity is supported by cross-cultural replication of the original factor models (Döpfner et al., 2014).

#### 2.3.3 Empathy

Different facets of empathy (Affective, Cognitive, Somatic, Positive, Negative) were assessed using the German version of the Cognitive, Affective, and Somatic Empathy Scales (CASES) (30 items, 0 = rarely, 2 = often) (Raine and Chen, 2018; Schleicher et al., 2021). The original English version of the CASES demonstrates good reliability and robust validity, including discriminant, incremental, and predictive aspects, as evidenced by meaningful associations with related constructs (Raine and Chen, 2018). The German version is currently undergoing further validation (Schleicher et al., 2021). The scale showed excellent internal consistency in the present sample (Cronbach’s *α* = 0.90).

The Saarbrueck Personality Questionnaire (SPF) (Paulus, 2009), the German version of the Interpersonal Reactivity Index, was used to assess four subscales: Perspective Taking, Fantasy, Empathic Concern, and Personal Distress (16 items, 1 = never, 5 = always). Overall, the questionnaire demonstrates good reliability as well as internal, external, and factorial validity (Paulus, 2009).

#### 2.3.4 Temperament and character

Personality facets were assessed using the Junior Temperament and Character Inventory (JTCI 12–18 R) (Goth and Schmeck, 2009), with the temperament domain comprising Novelty Seeking, Harm Avoidance, Reward Dependence, and Persistence, and the character domain including Self-Directedness, Cooperativeness, and Self-Transcendence (103 items, 0 = no, 4 = yes). The JTCI shows good reliability and robust validity, supported by consistent factor structures, criterion validity, and satisfactory agreement across raters and age versions (Goth and Schmeck, 2009).

#### 2.3.5 Physiological and affective reactivity

Heart rate was measured in beats per minute using a Movisens ECG and activity sensor EcgMove 4 (movisens GmbH, Karlsruhe, Germany). The sensor was attached non-invasively below the left chest. Prior to sensor placement, the skin was disinfected with an alcohol-based solution. The sensor remained in place for approximately 25 min and was removed after data collection. To assess participants’ momentary mood during the experiment, a numerical rating scale was administered at six predefined time points. Participants were asked to rate how they felt at that moment (“Right now, I feel…”) on a scale from 1 (not at all) to 10 (very much). The rating scale included four mood dimensions: Stress (“…stressed, tense, or overwhelmed.”), Anger (“…angry, annoyed, or furious”), Hostility (“…aggressive, hostile, or confrontational”), and Guilt (“…guilty, remorseful, or responsible”).

### 2.4 Procedure

At the beginning of the experiment, participants and, if under 18 years of age, their parents or legal guardians were informed about the study both verbally and in writing. Shortly thereafter, a standardized cover story was introduced, indicating that an opposing participant had just arrived. After addressing any remaining questions, written informed consent was obtained. Parents or legal guardians then left the room and completed the CBCL/6–18R.

The experimental session began with a baseline mood assessment and attachment of the heart rate sensor (0 min), followed by task instructions (see Supplementary material 1) and a second mood assessment (+5 min). Participants then assigned sealed envelopes containing tasks of varying difficulty to themselves and the alleged opponent. After a third mood assessment (+10 min), participants completed their assigned matrix reasoning tasks while the experimenter temporarily left the room.

Following task completion, a fourth mood assessment was conducted (+15 min), and participants completed additional questionnaires (DAF, AQ, YSR/11–18R, CASES, SPF, JTCI 12–18 R). The heart rate sensor was removed 5 min after completion of the DoQD (+20 min). At the end of the session, mood was assessed again before (+85 min) and after debriefing (+90 min), during which the cover story and study aims were explained. A credibility check was conducted, and all participants received €25. A detailed overview of the procedure is provided in Figure 1.

### 2.5 Data processing and statistical analysis

Heart rate data were processed using UnisensViewer (v1.16.46.0) and DataAnalyzer (v1.13.5; FZI, movisens GmbH, Karlsruhe, Germany). Heart rate (1-min output intervals) was averaged across four five-minute periods: baseline, task allocation (primary period of interest), task completion, and post-task, based on a detailed experimenter time log. Due to technical issues, heart rate data were unavailable for six participants, who were excluded from these analyses.

Missing questionnaire data were handled according to the respective scoring manuals. For six participants, CBCL data were incomplete or missing and were excluded from related analyses.

Statistical analyses were conducted using IBM SPSS Statistics 28 (IBM Corp., Armonk, NY). Due to non-normal data distribution, group differences were assessed using Mann–Whitney U and Kruskal–Wallis H tests. Changes over time in psychophysiological and affective measures were analyzed using Friedman tests with Wilcoxon signed-rank *post hoc* tests. Associations between the difficulty index and variables of interest were examined using Kendall’s tau correlations, with significant predictors entered into linear regression analyses. The significance level was set at 0.05. Effect sizes were interpreted according to Cohen’s conventional benchmarks (Cohen, 2013). To account for multiple comparisons in the correlational analyses, the False Discovery Rate (FDR) (Benjamini and Hochberg, 1995) procedure was applied. The FDR correction was applied separately within conceptually defined families of tests, comprising self-reported aggression (DAF, AQ, and YSR/11–18R), caregiver-reported aggression (CBCL/6–18R), empathy (CASES and SPF), and temperament and character (JTCI 12–18 R).

## 3 Results

### 3.1 Sample and descriptive statistics

**Figure 2:**
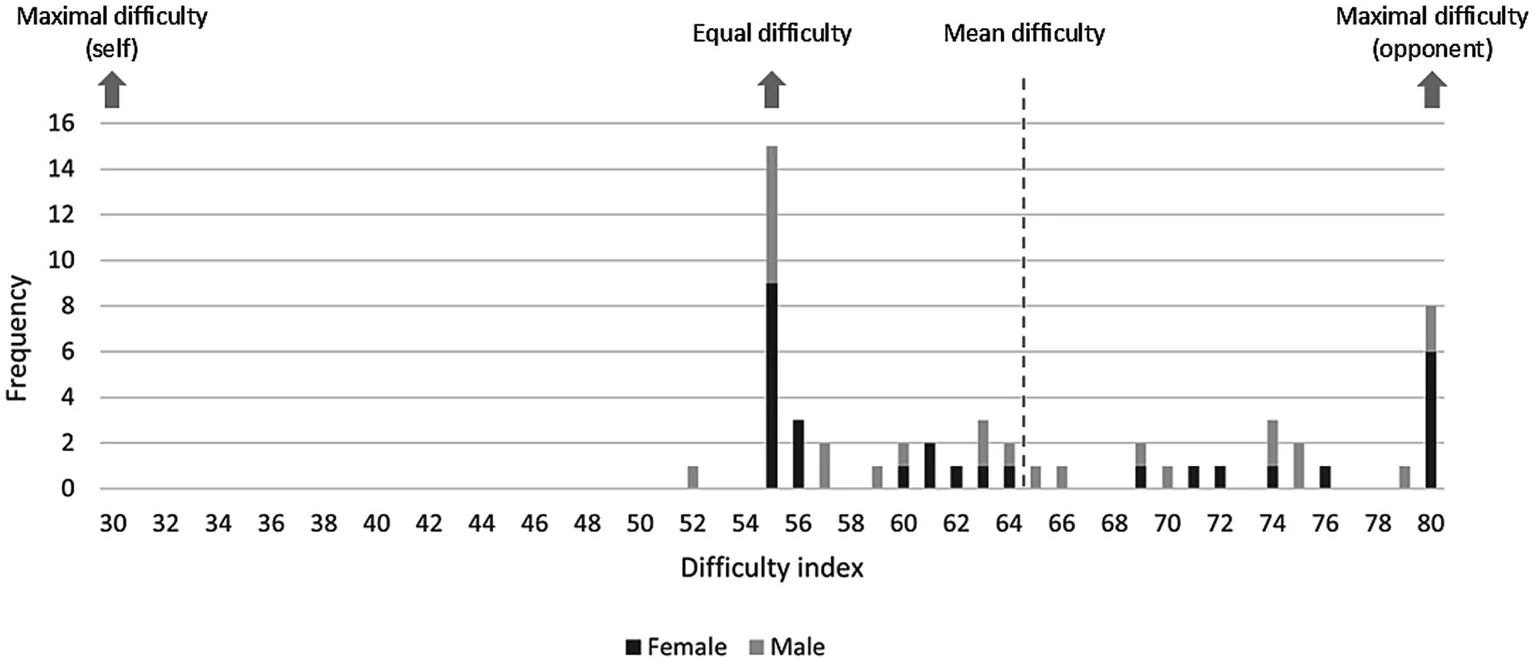
Frequency distribution of the difficulty index (female and male refer to biological sex). Bar heights indicate the number of participants at each observed difficulty index value. The dashed vertical line indicates the sample mean.

A total of 54 adolescents and young adults were included in the statistical analysis (biological sex: 29 female, 25 male; gender identity: 28 female, 26 male). Of these, 33 attended lower secondary school (Mittelschule), 2 attended intermediate secondary school (Realschule), 11 attended academic-track secondary school (Gymnasium), 4 were university students, and 4 were in other forms of education. Across all participants, the mean DoQD difficulty index was *M* = 64.59 (*SD* = 9.59; median = 62.50), with a possible range of 30 to 80 (actual range in the present sample: 52 to 80). For 15 participants (27.8%), the assigned difficulty index for the opponent was 55, corresponding to an equal allocation of task difficulty between themselves and the opponent, whereas 8 participants (14.8%) assigned the maximum possible difficulty index of 80 to the opponent. The distribution showed a skewness of 0.47 and a kurtosis of −1.33. An overview of the distribution of the difficulty index can be seen in Figure 2. On average, the participants solved their assigned tasks correctly at a rate of 87.59% (*SD* = 16.13%). The difficulty index did not differ between biological sexes (*U* = 361.50, *z* = −0.02, *p* = 0.986), gender identities (*U* = 351.50, *z* = −0.22, *p* = 0.827), or educational types (*H*(4) = 4.21, *p* = 0.379). There were also no correlations with age (τ = −0.12, *p* = 0.310). Regarding the credibility check, there was no difference in the difficulty index between participants who believed the cover story (*n* = 49) and those who were suspicious (*n* = 5; e.g., “There must be a catch somewhere”) (*U* = 87.00, *z* = −1.07, *p* = 0.283). Additional descriptive statistics for all main study variables are presented in Table 1.

**Table 1:** Descriptive statistics for the main study variables.

Variable	*M*	*SD*	Median	Range	*α*
Aggression					
DoQD difficulty index	64.59	9.59	62.50	52–80	–
DAF—anger-aggression	2.89	2.60	2.00	0–10	0.79
DAF—defensive aggression attribution	2.31	2.20	2.00	0–9	0.67
DAF—resource acquisition	0.74	1.15	0.00	0–5	0.48
DAF—exercise of power/dominance	1.54	1.85	1.00	0–7	0.63
DAF—reactive aggression	5.20	4.04	4.00	0–18	0.78
DAF—proactive aggression	2.28	2.52	1.00	0–8	0.67
AQ—physical aggression	14.76	4.73	14.00	9–30	0.76
AQ—verbal aggression	10.15	2.64	10.50	5–18	0.59
AQ—anger	13.78	3.69	13.00	7–23	0.67
AQ—hostility/distrust	15.48	4.38	15.00	8–25	0.74
CBCL—rule-breaking behavior	2.54	3.81	1.00	0–18	0.83
CBCL—aggressive behavior	3.81	4.24	2.00	0–16	0.85
CBCL—externalizing problems	6.35	7.69	3.00	0–33	0.91
YSR—rule-breaking behavior	3.67	3.24	3.00	0–11	0.72
YSR—aggressive behavior	5.35	4.31	4.00	0–17	0.80
YSR—externalizing problems	9.02	6.99	7.00	0–27	0.86
Empathy					
CASES—cognitive empathy	13.98	4.67	14.00	0–20	0.88
CASES—affective empathy	14.24	3.92	15.00	3–25	0.63
CASES—somatic empathy	10.63	4.15	11.00	2–19	0.74
CASES—negative empathy	19.24	5.54	19.70	2–29	0.74
CASES—positive empathy	19.61	5.82	21.00	2–28	0.83
SPF—fantasy	12.64	3.58	13.00	4–20	0.70
SPF—empathic concern	13.69	3.47	14.00	4–20	0.73
SPF—perspective taking	13.03	4.25	13.50	4–20	0.84
SPF—personal distress	10.35	3.51	10.00	4–19	0.71
Temperament and character					
JTCI—novelty seeking	27.57	8.41	26.50	9–51	0.71
JTCI—harm avoidance	26.12	11.03	27.50	1–46	0.74
JTCI—reward dependence	39.42	9.69	39.50	16–64	0.77
JTCI—persistence	32.09	10.02	30.50	3–56	0.87
JTCI—self-directedness	36.61	9.45	36.50	13–56	0.82
JTCI—cooperativeness	46.87	10.74	47.00	20–64	0.84
JTCI—self-transcendence	15.22	8.05	14.00	0–36	0.78

Ranges represent observed values. α, Cronbach’s alpha; DoQD, Degree-of-Quiz-Difficulty Paradigm; Reliability is not applicable to the DoQD paradigm. DAF, Differential Aggression Questionnaire; AQ, German Aggression Questionnaire; CBCL, Child Behavior Checklist; YSR, Youth Self-Report; CASES, Cognitive, Affective, and Somatic Empathy Scales; SPF, Saarbrueck Personality Questionnaire; JTCI, Junior Temperament and Character Inventory.

### 3.2 Associations of the difficulty index with psychological traits

With regard to prosocial variables, only one significant negative association emerged after FDR correction, namely between the difficulty index and self-reported scores on the JTCI Cooperativeness scale (*τ* = −0.29, *p* = 0.003). However, even when considering questionnaire measures alone, no significant correlations were found between self- and caregiver-reported aggression and self-reported empathy (all *p*s > 0.07). In contrast, caregiver-reported CBCL data showed positive associations with the difficulty index for Rule-Breaking Behavior (*τ* = 0.29, *p* = 0.009), Aggressive Behavior (*τ* = 0.23, *p* = 0.037), and, consequently the higher-order Externalizing Problems scale (*τ* = 0.24, *p* = 0.023), but no associations with Internalizing Problems (all *p*s > 0.107). In the sensitivity analysis, given *n* = 48 and *α* = 0.05 (two-tailed), the study had 80% power to detect correlations of *r* ≥ *0*.28 (equivalent to *τ* ≈ 0.18). The observed correlations between the difficulty index and caregiver-reported CBCL aggression scores exceeded this threshold, suggesting adequate sensitivity to detect effects of this magnitude. In the YSR, no relationship was found with the Social Desirability scale (*τ* = −0.07, *p* = 0.489). An overview of all correlations is provided in Table 2. Given the high correlation between the CBCL Rule-Breaking Behavior and Aggressive Behavior scales (*τ* = 0.53, *p* < 0.001), the higher-order Externalizing Problems scale, together with JTCI Cooperativeness, was included as predictors in the linear regression model. Demographic variables (age, sex, gender, school type) were excluded as predictors due to the absence of significant associations with the difficulty index. The regression model was significant (*F*(2, 45) = 7.53, *p* = 0.002), explaining 25% of the variance. Cooperativeness was a significant negative predictor (*β* = −0.28, *p* = 0.039), and the Externalizing Problems scale was a significant positive predictor (*β* = 0.37, *p* = 0.007) (Table 3). As a robustness check, the central correlational analyses were repeated after excluding the eight participants who assigned the maximum possible difficulty index (80). The overall pattern of results remained unchanged, although the association with the CBCL Aggressive Behavior scale was no longer statistically significant.

**Table 2:** Correlations between difficulty index and psychological traits.

Trait/Scale	τ	*p*	*p*_FDR_	*n*
Aggression				
DAF—anger-aggression (reactive)	0.15	0.141	0.994	54
DAF—defensive aggression attribution (reactive)	0.00	0.994	0.994	54
DAF—resource acquisition (proactive)	0.05	0.637	0.994	54
DAF—exercise of power/dominance (proactive)	−0.09	0.401	0.994	54
DAF—reactive aggression	0.13	0.214	0.994	54
DAF—proactive aggression	−0.06	0.549	0.994	54
AQ—physical aggression	0.03	0.767	0.994	54
AQ—verbal aggression	−0.05	0.625	0.994	54
AQ—anger	0.08	0.452	0.994	54
AQ—hostility/distrust	−0.07	0.466	0.994	54
**CBCL**—**rule-breaking behavior**	**0.29**	0.009*	**0.027**	**48**
**CBCL**—**aggressive behavior**	**0.23**	0.037*	**0.037**	**48**
**CBCL**—**externalizing problems**	**0.24**	0.023*	**0.035**	**48**
YSR—rule-breaking behavior	0.02	0.872	0.994	54
YSR—aggressive behavior	−0.01	0.945	0.994	54
YSR—externalizing problems	0.01	0.964	0.994	54
Empathy				
CASES—cognitive empathy	−0.19	0.058	0.139	54
CASES—affective empathy	−0.10	0.307	0.345	54
CASES—somatic empathy	−0.16	0.112	0.144	54
CASES—negative empathy	−0.18	0.077	0.139	54
CASES—positive empathy	−0.16	0.100	0.144	54
**SPF**—**fantasy**	**−0.20**	**0.048**	**0.139**	**54**
**SPF**—**empathic concern**	**−0.21**	**0.038**	**0.139**	**54**
SPF—perspective taking	−0.18	0.074	0.139	54
SPF—personal distress	−0.06	0.552	0.552	54
Temperament and character				
JTCI—novelty seeking	−0.04	0.677	0.699	54
JTCI—harm avoidance	0.04	0.672	0.699	54
JTCI—reward dependence	−0.14	0.150	0.350	54
JTCI—persistence	−0.04	0.688	0.699	54
JTCI—self-directedness	0.04	0.699	0.699	54
**JTCI**—**cooperativeness**	**−0.29**	0.003*	**0.021**	**54**
JTCI—self-transcendence	−0.15	0.133	0.350	54

DAF, Differential Aggression Questionnaire; AQ, German Aggression Questionnaire; CBCL, Child Behavior Checklist; YSR, Youth Self-Report; CASES, Cognitive, Affective, and Somatic Empathy Scales; SPF, Saarbrueck Personality Questionnaire; JTCI, Junior Temperament and Character Inventory. * indicates that the result remains significant after FDR correction. p_FDR **=**_ Benjamini–Hochberg false discovery rate-adjusted *p* value. Significant correlations are marked in bold font.

**Table 3:** Regression analysis predicting the difficulty index.

Dependent variable	Predictors	*B*	SE	95% CI *B*		*β*	*t*	*p*	*R* ^2^	Adj. *R*^2^
				Lower bound	Upper bound					
Difficulty index	**Externalizing problems**	**0.45**	**0.16**	**0.13**	**0.77**	**0.37**	**2.83**	**0.007**	0.25	0.22
	**Cooperativeness**	**−0.25**	**0.12**	**−0.48**	**−0.01**	**−0.28**	**−2.13**	**0.039**		

Significant predictors are marked in bold font.

### 3.3 Associations of the difficulty index with psychophysiological and affective responses

**Figure 3:**
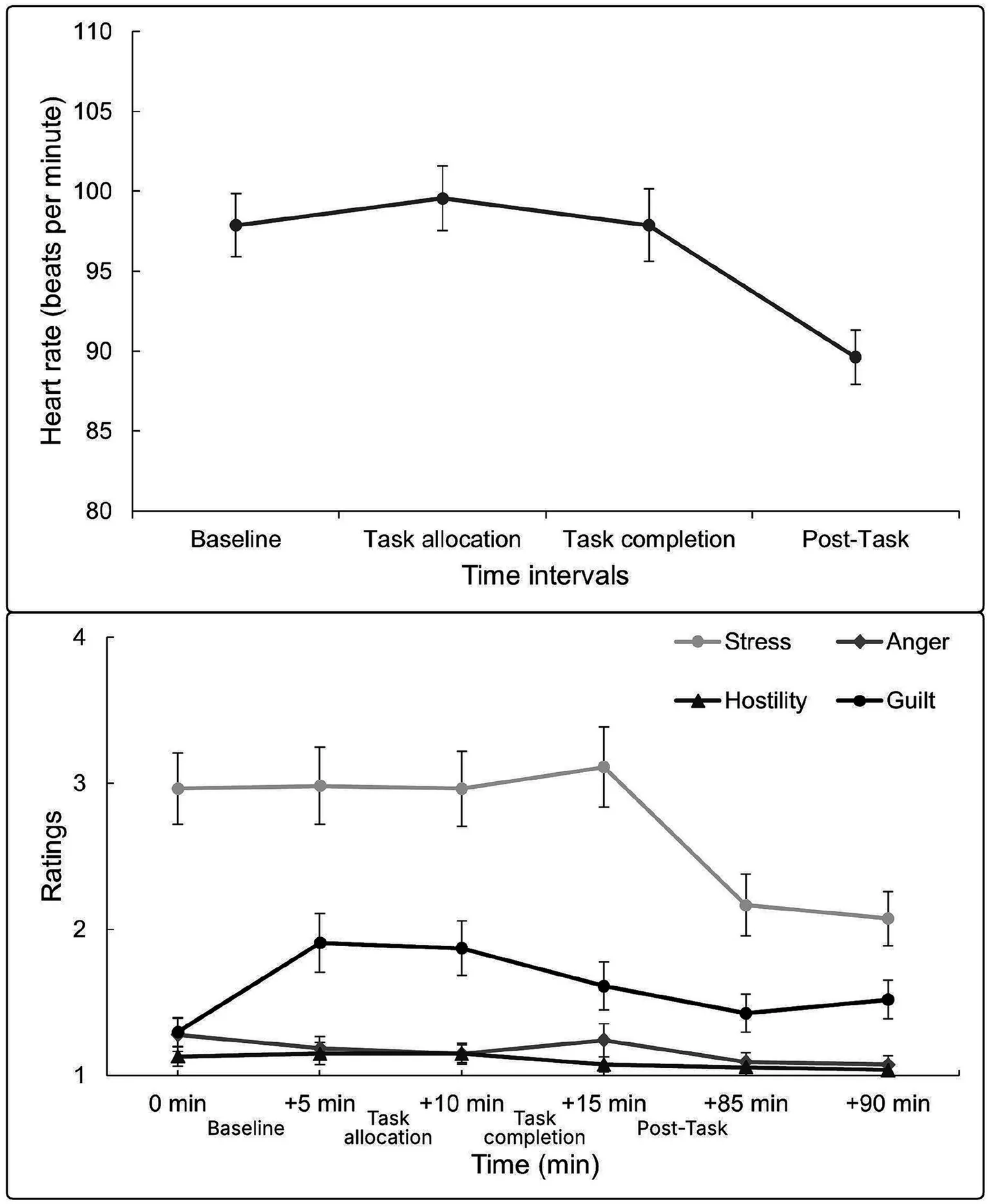
Changes in psychophysiological and affective variables over time (error bars indicate standard errors of the mean).

Significant time effects were observed for the mood scales Stress (*χ*^2^(5) = 51.64, *p* < 0.001), Anger (*χ*^2^(5) = 14.27, *p* = 0.01), and Guilt (*χ*^2^(5) = 22.31, *p* < 0.001), as well as for heart rate (*χ*^2^(3) = 62.45, *p* < 0.001) (Figure 3). Regarding associations with the difficulty index, significant correlations were found only with the Guilt scale at time points +10 min (after dividing the questions between the participant and the opponent; *τ* = 0.24, *p* = 0.028), +15 min (after completing the participant’s own tasks; *τ* = 0.34, *p* = 0.003), and +90 min (after debriefing concerning the cover story; *τ* = 0.32, *p* = 0.005). With regard to changes over time, pairwise Wilcoxon signed-rank tests on the Guilt scale (after FDR correction) revealed only one significant time difference: scores at +5 min (after the instruction and before the allocation of quiz questions) were significantly higher than at 0 min (after the start of the experiment and before the instruction) (*Z* = −3.11, *p* = 0.002, *r* = 0.42). No significant correlations between the difficulty index and the other mood ratings (all *p*s > 0.061) or heart rate (all *p*s > 0.419) were identified.

## 4 Discussion

The present study aimed to develop and provide preliminary validity evidence for the Degree-of-Quiz-Difficulty Paradigm (DoQD) as a behavioral paradigm relevant to the assessment of proactive aggression in adolescents and young adults. Proactive aggression was operationalized as assigning higher task difficulty to an alleged opponent in a competitive, goal-directed context. We expected the resulting difficulty index to be positively associated with self- and caregiver-reported aggression, particularly proactive components, and negatively associated with empathy-related and prosocial traits. Mood and heart rate changes were explored, with no expected associations with psychophysiological stress responses. Overall, the findings provided mixed support for the preliminary validity of the DoQD, with evidence supporting some, but not all, of the hypothesized associations.

Descriptively, participants predominantly chose either equal or maximally unfair difficulty allocations, assigning the highest difficulty to the opponent. No significant differences in the difficulty index emerged with respect to sex, gender, educational type, or age. While there is no robust evidence for differences in aggression across education types, previous research suggests that proactive aggression tends to remain relatively stable from late childhood into adolescence (Guo et al., 2024; Vaughan et al., 2024). Unexpectedly, no gender differences emerged, although boys typically report higher levels of aggression across studies (Mayberry and Espelage, 2007; Guo et al., 2024). This finding may be explained by the fact that the present paradigm captures a more covert, indirect form of proactive aggression, which has been reported to be more prevalent in females (Hess and Hagen, 2006).

With regard to discriminant validity, the difficulty index showed a negative association with the prosocial trait cooperativeness, reflecting tendencies toward collaboration and helpfulness. However, after applying FDR correction, associations with other empathy scales were no longer significant. While empathy is often found to be negatively associated with aggression, previous research has yielded mixed findings, with studies reporting links between empathy deficits and aggression, impulsivity, and violence (Blair, 2018; Deschamps et al., 2018), and others indicating weak, absent, or even positive associations (Vachon et al., 2014; Kahhale et al., 2024). These mixed findings may partly reflect heterogeneity in the operationalization of empathy and aggression, as well as insufficient differentiation of their subtypes (Vachon et al., 2014; Euler et al., 2017). In line with this, the present results provide only limited support for an association between proactive aggression and empathy, while the observed link with cooperativeness is consistent with prior research on prosocial traits (Fossati et al., 2009; Euler et al., 2017; Kerekes et al., 2017). Overall, these findings provide partial support for the discriminant validity of the DoQD.

With regard to convergent validity, the findings were mixed. Contrary to our initial hypotheses, the difficulty index was not significantly associated with self-reported proactive aggression. Notably, some previous studies have reported similar patterns (e.g., Boccadoro et al., 2021), suggesting that associations between self-reported proactive aggression and objective behavioral indicators may not always emerge in experimental paradigms. Instead, we found that caregiver-reported externalizing behaviors (specifically rule-breaking and aggressive behavior) significantly predicted the difficulty index. In other words, the higher caregivers rated their child’s externalizing and aggressive behavior, the higher the level of difficulty assigned to the alleged opponent. Although the respective scales do not explicitly distinguish between reactive and proactive aggression, the Rule-Breaking Behavior scale appears to reflect more proactive components and showed comparatively stronger associations with the difficulty index. A closer examination of the item content supports this interpretation: whereas the Aggressive Behavior scale includes more reactive elements such as mood swings and temper outbursts, the Rule-Breaking Behavior scale emphasizes planned and instrumental behaviors (e.g., lying, stealing, deliberate destruction of property). This interpretation is consistent with findings by Raine et al. (2006), who reported significant positive associations between both reactive and proactive aggression and parent-rated aggressive behavior on the CBCL, as well as a specific association between parent-rated rule-breaking behavior and proactive—but not reactive—aggression. Similar results were reported by Scarpa et al. (2010), who found that caregiver-reported rule-breaking behavior was significantly associated with proactive aggression. At the same time, our data also indicate that a clear differentiation between reactive and proactive components remains challenging, as correlations with the difficulty index were of comparable magnitude across scales, suggesting conceptual overlap in their operationalization. Nevertheless, it is important to note that the DoQD did not induce provocation or frustration, making a predominantly proactive interpretation of the measured behavior plausible. Discrepancies between self- and caregiver-reports may further reflect differences in evaluative standards. Whereas parents tend to base their judgments on broader societal norms, adolescents often evaluate their behavior relative to their peers, potentially leading to more favorable self-assessments (Schomaker et al., 2015; Roos et al., 2016). In addition, the validity of self-reports depends on adolescents’ capacities for self-perception and self-reflection (Jurkowski and Hänze, 2014). The divergence between self- and caregiver-reports may therefore partly reflect biased self-perception. Although no association emerged with the YSR Social Desirability scale, potential effects of socially desirable responding should still be considered. Moreover, externalizing behaviors, by definition, are more observable and impactful for others than for the individual themselves, which may enhance their detectability in informant reports. Taken together, these findings provide preliminary support for convergent validity based on caregiver-reported externalizing behaviors, whereas no corresponding evidence emerged for self-reported proactive aggression.

Exploratory analyses revealed changes over time in perceived stress, guilt, and heart rate. Stress ratings and heart rate were elevated at the beginning of the experiment and during the completion of the matrix tasks but decreased immediately afterward. The difficulty index was not significantly associated with subjective stress ratings or heart rate changes, suggesting that temporal fluctuations in physiological and subjective arousal were more likely attributable to general task demands (e.g., novelty of the experimental situation and performance-related demands) rather than emotional reactions related to proactive aggression. This finding supports our assumption that, in contrast to reactive aggression, proactive aggression is characterized by lower psychophysiological arousal, likely reflecting greater emotional control (Blair, 2018). Previous studies have similarly reported reduced stress reactivity or even hypoarousal in response to threat (Bobadilla et al., 2012; Thomson et al., 2021).

In contrast, significant positive associations emerged between the difficulty index and feelings of guilt. Guilt increased significantly after the instruction and prior to the allocation of tasks, possibly reflecting anticipatory awareness of the upcoming decision and the responsibility associated with being selected to determine task difficulty. Notably, higher difficulty indices were associated with stronger feelings of guilt following task allocation, which persisted after completion of the quiz and increased again after the final debriefing (when participants were informed that no real opponent existed and that their allocation decisions constituted the central focus of the study). Taken together, these findings suggest that proactive aggressive behavior may be accompanied by feelings of guilt. However, such feelings do not appear to exert a protective effect in preventing the aggressive act. Instead, the anticipated personal gain may outweigh the experienced guilt, which is seemingly accepted as a trade-off and may intensify once the behavior is disclosed. Previous research has mainly focused on trait-like tendencies toward guilt and shame in relation to proactive aggression, generally reporting negative associations (Olthof, 2012; Broekhof et al., 2021). In contrast, we assessed guilt as a state variable, capturing its dynamic emergence during aggressive behavior. Future studies should therefore consider not only general dispositions toward guilt and shame but also their temporal dynamics, as these emotions may occur during aggressive acts without necessarily preventing them.

An important consideration in interpreting the present findings is that assigning more difficult quiz questions to the opponent simultaneously increased the participant’s own probability of winning. Consequently, the current design cannot fully disentangle proactive aggression from strategic competitive decision-making or reward maximization. The DoQD should therefore be interpreted as a behavioral paradigm with relevance to the assessment of proactive aggression rather than as a definitive measure of the construct. Further validation studies are needed to establish its discriminant validity with respect to related competitive decision-making processes.

Several limitations should be considered. First, some of the expected effects did not emerge, which may be related to the relatively small sample size of this initial validation study. Although the sample met the requirements indicated by the sensitivity and *a priori* power analysis, the study may still have been underpowered to detect smaller effects. Second, assigning more difficult tasks to the opponent simultaneously increased the participant’s own probability of winning. Consequently, the current design cannot fully disentangle proactive aggression from strategic competitive decision-making or reward maximization. Future studies should further examine the discriminant validity of the DoQD with respect to these related constructs. Third, the additional €10 reward may have varied in subjective value, potentially affecting instrumental motivation. Fourth, although validated instruments were used, the assessment of aggression relied on specific questionnaires, which may have influenced the pattern of associations. Moreover, some of the scales and subscales showed relatively low internal consistency in the present sample, which may have attenuated associations involving these measures and should be considered when interpreting the findings. Future studies may benefit from including alternative measures of reactive and proactive aggression, such as the Reactive–Proactive Aggression Questionnaire (RPQ) (Raine et al., 2006), and from assessing related constructs (e.g., empathy) using also informant reports. Finally, the study was conducted in a non-clinical sample recruited exclusively in Germany. Future research should extend the paradigm to clinical or high-risk populations, broader age ranges, and different cultural contexts to enhance variability and generalizability.

Taken together, the present findings provide preliminary support for the DoQD as a behavioral paradigm relevant to the assessment of proactive aggression, while also highlighting the need for further validation. Following further validation, including studies examining its discriminant, criterion-related, and incremental validity, as well as applications in clinical samples, the Degree-of-Quiz-Difficulty Paradigm may represent a promising, time- and cost-efficient tool. It provides a more differentiated behavioral measure of aggressive decision-making and captures proactive aggression in the absence of provocation as well as prosocial or even altruistic choices (e.g., assigning oneself more difficult tasks). By reducing reliance on self- or informant-reports, the paradigm may help mitigate reporting biases. Overall, the paradigm appears to meet the four key criteria of proactive aggression tasks: (1) the behavior is driven by instrumental motivation to obtain an incentive, (2) it involves a moral decision component, (3) it occurs without anger-related arousal, and (4) it takes place in the absence of provocation (Zhu et al., 2019). Moreover, due to its standardized structure and low administrative burden, the paradigm may be suitable for clinical settings and digital implementation. In the assessment of children, adolescents, and young adults, it may serve as a complementary behavioral measure alongside established self- and informant-report instruments, thereby broadening the multimethod assessment of proactive aggression. Its competitive framework also enables conceptual comparability with established reactive aggression paradigms (e.g., modified versions of the Taylor Aggression Paradigm), facilitating combined assessments of reactive and proactive aggression within a unified context.

## Data Availability

The raw data supporting the conclusions of this article will be made available by the authors, without undue reservation.

## Supplementary material

Supplementary Material 1 Paradigm instructions.
