# Supplementary material for: Measuring proactive-instrumental aggression in adolescents and young adults—development and preliminary validity evidence for the Degree-of-Quiz-Difficulty Paradigm
Source: Front Psychol. 2026 Sep 3;17:1876347. doi: 10.3389/fpsyg.2026.1876347 (PMC13581552; doi:10.3389/fpsyg.2026.1876347)
Supplement: Supplementary file 1 [file Data_sheet_1.pdf]

Supplementary Material 1. Paradigm instructions.

"You will now compete against the other person in a quiz duel. Each of you has to answer 10 questions, which vary in difficulty. The difficulty levels are indicated on the envelopes, where 1 means 'very easy' and 10 means 'very difficult'. However, you are not allowed to see the questions yet. You must therefore not open the envelopes until I tell you to do so.

For each correctly answered question, you receive one point. Thus, a maximum of ten points can be achieved. The person who answers more questions correctly wins and will receive an additional ten-euro voucher.

However, we have included a small trick. Before you arrived, you were randomly selected to be allowed to distribute the tasks between yourself and the other person. This means that you may decide how difficult your own questions and the questions for the other person will be. The other person will, of course, not be informed that you assigned the questions. This will remain between us.

You now have three minutes to distribute the tasks. You should place the sealed envelopes containing your tasks into the left box and the sealed envelopes containing the tasks you want to give to the other person into the right box. I will not see which questions you assigned to the other person or how difficult they are, as I will hand over the other person's box while it is closed.

Remember that the person who answers more questions correctly wins. You may now distribute the envelopes. I will collect the boxes again in three minutes and prepare everything else in the meantime."
